# KCNE gene family: From basic functions to diseases

**DOI:** 10.1016/j.gendis.2025.101918

**Published:** 2025-11-05

**Authors:** Junshen Xiao, Xu Cheng, Dou Huang, Shichao Wei, Zhaoyang Hu

**Affiliations:** a Department of Anesthesiology, West China Hospital, Sichuan University, Chengdu, Sichuan 610041, China; b Laboratory of Anesthesia and Critical Care Medicine, National-Local Joint Engineering Research Centre of Translational Medicine of Anesthesiology, West China Hospital, Sichuan University, Chengdu, Sichuan 610041, China

**Keywords:** KCNE, MiRP, Multiorgan, Potassium channel, Review

## Abstract

The KCNE single-span transmembrane subunits modulate the function of voltage-gated potassium (K_v_) channels, which are critical for maintaining electrical excitability and signal transduction across various tissues, by interacting with different K_v_ channel α-subunits to form diverse channel complexes with unique biophysical properties and regulatory mechanisms. These interactions profoundly impact physiological processes. Mutations or altered expression of KCNE genes have been implicated in a range of pathophysiological conditions. Emerging research has provided significant insights into the dynamic interactions between the KCNE gene and K_v_ channels, highlighting their critical roles in channel function modulation. This increasing understanding paves the way for designing novel therapeutic strategies to address ion channel-related disorders. To deepen the understanding of research advancements, this review seeks to elucidate the roles of KCNE across various organs and to provide constructive recommendations for future research.

## Introduction

The KCNE family is a group of small transmembrane auxiliary subunits that play critical roles in regulating the function of voltage-gated potassium (K_v_) channels.^1^ The KCNE gene family comprises six regulatory subunits (KCNE1–5 and the recently identified KCNE6 isoform) characterized by a conserved single transmembrane helix structure. These auxiliary subunits profoundly modulate the functional expression of multiple ion channel families, most classically K_v_ channels, through coordinated regulation of protein trafficking kinetics, ion flux velocity, voltage sensitivity thresholds, and pore selectivity filters, with these shared principles extending to structurally divergent channels, including anoctamins and hyperpolarization-activated cyclic nucleotide-gated (HCN) channels.^2^, ^3^, ^4^, ^5^, ^6^, ^7^ Their capacity to alter channel activation kinetics directly impacts the temporal dynamics of potassium efflux, whereas shifts in voltage-dependent gating determine the membrane potential required for channel opening. Furthermore, structural remodeling of the pore architecture enables the selective permeation of specific ions. This multidimensional regulation emerges from dynamic interactions between KCNE proteins and K_v_ channel α-subunits, allowing precise tuning of electrical signaling pathways in excitable tissues.^8^^,^^9^

In addition to their structural roles in channel assembly, KCNE subunits (including KCNE1-encoded MinK and MiRP isoforms) cooperate with α-subunits to orchestrate systemic physiological regulation. Their tissue-specific interactions with diverse K_v_ complexes enable essential functions ranging from action potential modulation to endocrine homeostasis. However, this functional diversity comes with clinical consequences: mutations disrupting KCNE-mediated regulation manifest as multiorgan pathologies affecting cardiac rhythm, neuronal excitability, or electrolyte balance.^10^, ^11^, ^12^, ^13^

While recent advances have elucidated these molecular mechanisms, a systematic synthesis of the organ-specific regulatory networks of KCNE1–6 is lacking. To further understand the research progress in this area, this review aims to elucidate the important roles of KCNE genes in various organs and to offer constructive suggestions for further research.

## KCNE family and diverse ion channels

The KCNE gene family (KCNE1–6) encodes auxiliary subunits known as MinK and related peptides (MiRPs), which are single-pass transmembrane proteins that are 103–177 residues in length.^7^^,^^9^ These proteins exhibit highly conserved modular structural features: an extracellular N-terminal domain containing glycosylation sites that regulate membrane localization, a central transmembrane helix that mediates conformational coupling with primarily K_v_ α subunits, and an intracellular C-terminal domain that integrates intracellular signaling through phosphorylation motifs.^14^ K_v_ α subunits, as the core components of voltage-gated potassium channels, adopt a six-transmembrane topology (S1–S6) formed by four homologous domains. The S4 segment contains charged arginine residues that constitute the voltage sensor, whereas the S5–S6 helices collectively form the ion-conducting pore.^15^ KCNE proteins can precisely regulate the mechanical trajectory of the S4 segment by allosterically coupling their transmembrane regions to the voltage-sensing domains (S1–S4) of α-subunits. N-terminal acidic residues modulate the displacement amplitude of the S4 helix via electrostatic interactions, and C-terminal phosphorylation dynamically adjusts channel inactivation kinetics.^16^^,^^17^ This multidomain coordinated action enables KCNE proteins to act as allosteric modulators, dynamically altering channel activation thresholds and gating rates. Their mechanisms include optimizing ion selectivity by reshaping pore geometry, recalibrating activation curves by adjusting voltage sensor sensitivity, and enabling responsiveness to physiological stimuli through coupling with intracellular second messengers. Structural divergences among KCNE subtypes, such as the negatively charged residues of the N-terminus of the KCNE3 protein or the unique C-terminal glycosylation pattern of the KCNE4 protein, further confer tissue-specific regulatory capacities.^18^^,^^19^

Besides, KCNE subunits demonstrate analogous regulatory potential for functionally distinct ion channels. Phosphorylation of the KCNE1 β-subunit may enable transmembrane protein 16A (TMEM16A), a calcium-activated chloride channel vital for epithelial secretion, to acquire angiotensin II responsiveness, thereby converting its gating modality from calcium-dependence to voltage-dependent Cl^−^ conduction.^20^ While KCNE2-encoded protein MiRP1 elevates HCN4 membrane abundance and hyperpolarization-activated current, which is related to funny current (If) in cardiac pacemakers, likely via stabilization of HCN4 complexes or chaperone-assisted trafficking, the exact mechanisms require further resolution.^21^^,^^22^

## Functions of the KCNE family in different organs or systems

### Heart

As a β-subunit that regulates potassium ion channels, KCNE primarily plays a role in modulating cardiac electrophysiology in the heart. Cardiac potassium currents play crucial roles in maintaining cardiac electrophysiological stability and normal heart rhythm.^23^ The primary potassium currents in the heart include the transient outward potassium current (Ito), the inward rectifier potassium current (IK1), the delayed rectifier potassium current (IKr and IKs), and the acetylcholine-regulated potassium current (IKACh), whereas KCNE subunits are associated mainly with the regulation of IKr and IKs.^23^ Delayed rectifier potassium current (IKs) is composed of K_v_7.1 and MinK subunits. The current formed by K_v_7.1 alone features faster activation and inactivation kinetics but with a smaller amplitude. When the KCNE1-encoded protein MinK co-assembles with K_v_7.1 channels, it markedly delays IKs activation kinetics and prolongs cardiac repolarization by slowing voltage sensor movement and reducing open probability. MinK prolongs the opening time of the K_v_7.1 channel and slows down the activation and inactivation dynamics, thereby increasing the stability and sustainability of the current.^24^ During cardiac repolarization, the IKs current works in conjunction with other potassium currents, such as IKr, to ensure the proper termination of the action potential. When the heart rate increases, the IKs current can rapidly increase, thereby shortening the action potential duration and preventing excessively prolonged action potentials that could lead to arrhythmias.^25^ While the hERG current, which conducts IKr, remains the primary target regulated by KCNE2-encoded protein MiRP1 in the heart, MiRP1 reduces the hERG current amplitude and accelerates its inactivation during heart repolarization.^26^ Numerous mutations and polymorphisms associated with arrhythmias have been identified in genes encoding cardiac ion channels. Inherited arrhythmias predominantly arise from impaired myocardial repolarization and prolonged action potential duration due to reduced potassium efflux through defective IKr or IKs channels, manifesting as prolonged QT intervals on surface electrocardiograms and significantly increasing the risk of torsades de pointes, ventricular fibrillation, syncope, and sudden cardiac death.^27^ Long QT syndrome types 5 (LQT5) and 6 (LQT6) are caused by mutations in KCNE1 and KCNE2, respectively. These auxiliary subunits disrupt cardiac electrical stability by altering channel gating properties or membrane trafficking.^26^ The IKs channel, formed by the α-subunit K_v_7.1 and auxiliary subunit MinK, exhibits loss-of-function mutations that reduce the repolarizing IKs current amplitude, prolonging the action potential duration and QT intervals. Under physiological conditions, β-adrenergic receptor-PKA signaling activated by sympathetic stimulation phosphorylates K_v_7.1-MinK channels to increase their activity and shorten the action potential duration.^28^ However, these mutations impair cAMP-dependent regulatory capacity without interfering with PKA-mediated phosphorylation, rendering the channels unresponsive to heart rate acceleration.^29^ Critically, K_v_7.1 and MinK traffic via distinct pathways to the surface sarcolemma, where they assemble into functional channels, a delayed surface assembly process essential for maintaining IKs as a repolarization reserve. This assembly mechanism involves microtubule plus-end binding protein EB1, which preferentially binds to dimeric K_v_7.1 to promote surface delivery^30^; LQT1-associated mutations (*e.g.*, Y111C) disrupt EB1 binding, impairing channel trafficking and reserve function.^31^ Furthermore, MinK increases Kv7.1 sensitivity to phosphatidylinositol 4,5-bisphosphate (PIP2) 100-fold through specific binding, stabilizing channel open states, and delaying inactivation. LQT5-associated mutations, such as C-terminal mutations or truncations, markedly reduce PIP2 binding affinity, accelerating channel inactivation and diminishing adaptive responses to heart rate variations.^32^ These multilevel regulatory defects collectively contribute to insufficient repolarizing currents. The reported cardiac arrhythmias associated with mutations in KCNE genes are summarized in Table 1.

**Table 1:** Cardiac arrhythmia associated with mutations in KCNE genes.

KCNE	Disease	Mutation	Effects of KCNE	References
KCNE1	LQT5	A8V	Revealed marked bradycardia and QT interval prolongation in the electrocardiogram	116
		R98W	Revealed significant bradycardia with QT prolongation during exercise and reduced IKs currents in electrophysiological analyses	116
		R32H	Decreased IKs current amplitude equivalent to 78 % without any changes in gating	117
		V47F	Decreased IKs current and altered gating	118
		W87R		
		G52R	Decreased IKs current to 50 %	119
		S74L	Decreased IKs current by shifting the voltage dependence of activation and accelerating channel deactivation	120
		Y81C	Decreased IKs current and enhanced the effect of IKs activator	121
		V109I	Decreased IKs current by 36 %	122
		D76N	Decreased IKs current and exhibited a strong dominant-negative effect, with a higher risk of delayed cardiac repolarization and arrhythmia	120
		D85N	Prolonged action potential duration in KCNE1–D85N-containing induced pluripotent stem cell-derived cardiomyocytes	123
		G38S	Prolonged QT interval under conditions of hypokalemia and hypomagnesemia	124,125
		R36H	Induced a 47 % reduction in IKs current with a transient QT prolongation phenotype	126
	Atrial fibrillation	G60D	Showed a gain-of-function of IKs current	127
		G25V		
		G38S	Reduced IKs current and decreased membrane expression of KCNQ1	128
KCNE2	LQT6	T8A	Showed slower activation, faster deactivation, and increased drug sensitivity	4
		Q9E		
		T10M	Reduced Ikr current and exacerbated by auditory stimuli or electrolyte disturbances	34
		M54T	Increased drug-induced LQTS sensitivity	4,33
		I57T	Increased drug-induced LQTS sensitivity	4,33
		V65M	Reduced IKs current with accelerated inactivation	129
		I20N	Increased susceptibility to long QT syndrome in a neonate	130
		R27H		
	Atrial fibrillation	R27C	Had a gain-of-function effect on the KCNQ1–KCNE2 channel	131
KCNE3	LQTS	T4A	Decreased current of KCNQ1/KCNE3 complex	132
		R99H		
KCNE4	Atrial fibrillation	E145D	Had an association with the atrial fibrillation phenotype in the Chinese population	133
KCNE5	Atrial fibrillation	L65F	Showed a significant concentration-dependent gain of function in IKs current	48
	Brugada syndrome	Y81H	Had a gain-of-function effect on Ito in male patients	47
		D92E		
		E93X		

Unlike KCNE1’s primary role in IKs regulation, KCNE2 critically modulates the rapid delayed rectifier potassium current (IKr) through hERG channels. MiRP1 suppresses the hERG current amplitude while accelerating its inactivation kinetics, profoundly influencing repolarization.^4^ Given the high susceptibility of hERG to pharmacological blockade, certain KCNE2 polymorphisms (*e.g.*, T8A, Q9E) increase the drug sensitivity of hERG–MiRP1 complexes, explaining the elevated risk of drug-induced LQTS (type 6) or hypokalemia-associated arrhythmias in carriers.^33^^,^^34^ Beyond potassium channel regulation, KCNE subunits critically modulate HCN channels that conduct the cardiac funny current (If). MiRP1 co-assembles with HCN4 in sinoatrial node cardiomyocytes, accelerating If activation kinetics and enhancing diastolic depolarization to stabilize heart rate during adrenergic stimulation. This regulation is physiologically distinct from KCNE-mediated Kv current modulation, as HCN channels contribute to pacemaker automaticity rather than repolarization.^21^^,^^22^ Despite established associations between KCNE2 and congenital/acquired LQTS, species differences complicate mechanistic studies. Critically, reduced slow-delayed rectifier potassium currents (*e.g.*, IKr, IKs) typically prolong cardiac repolarization by diminishing net repolarizing flux, a fundamental principle observed in human LQTS pathologies. However, murine ventricular cardiomyocytes evade this outcome through evolutionary adaptation: they lack functional IKr (hERG/MiRP1-dependent) and exhibit minimal IKs due to attenuated K_v_7.1 expression, yet paradoxically maintain abbreviated action potentials. This apparent contradiction is resolved by abnormalities of Ca^2+^ currents or compensatory overexpression of rapid-activating potassium currents such as IKur and Ito, which generate more outward currents than in humans, thereby dominating repolarization.^35^, ^36^, ^37^ Consequently, the absence of physiological slow-rectifier dependence severely limits murine model extrapolation to human hERG–MiRP1/K_v_7.1–MiRP1 regulatory mechanisms. Furthermore, KCNE subunits exhibit differential regulation of transient outward currents. MiRP2 strongly suppresses K_v_4.3-mediated currents in heterologous systems, reducing amplitude while decelerating activation, inactivation, and recovery kinetics; notably, K_v_4.3 underlies critical repolarizing currents to cardiac Ito.^38^ Conversely, KCNE4 expression enhances repolarizing current density by augmenting both Ito (K_v_4.2-dependent) and IKur (K_v_1.5-dependent), functionally paralleling ventricular KCNE2 roles in murine models.^39^ Given the unresolved *in vivo* protein interactions and compensatory current adaptations in systems, the precise physiological significance of KCNE3 and KCNE4 in cardiac electrophysiology warrants detailed mechanistic investigation.

The multifaceted roles of KCNE family members in cardiac repair and electrophysiological regulation further reveal their therapeutic potential and complexity. In gene therapy, adenoviral KCNE3 delivery to guinea pig left ventricles significantly shortens the action potential duration, suggesting novel intervention strategies for LQTS.^40^ Post-myocardial infarction heart failure is associated with KCNE2 down-regulation, ventricular dilation, and fibrosis. Sacubitril/valsartan (LCZ696) improves cardiac function by increasing KCNE1/2 expression, suggesting its therapeutic potential.^41^^,^^42^ Additionally, drug-induced QTc prolongation is a multifactorial phenomenon involving numerous medications that affect ion channels (including KCNE-regulated potassium channels).^43^^,^^44^ Case reports indicate that the KCNE1–D85N mutation may predispose patients to drug-induced LQTS. Specifically, the common anesthetic propofol inhibits the IKs channel in patients with this mutation, potentially causing unexpected severe adverse cardiac events during anesthesia, indicating the importance of genotyping and phenotyping to individualize drug therapy.^45^^,^^46^ As an X-linked gene, KCNE5 remains understudied, with current evidence linking its variants to atrial fibrillation and Brugada syndrome rather than LQTS.^47^^,^^48^ European male cohorts show associations between KCNE5 noncoding mutations, shortened PR intervals, and elevated atrial fibrillation risk.^49^ Animal models support this regulatory role: ventricular MiRP4 maintains electrical stability through K_v_2.1 current modulation in male mice, and its deletion enhances potassium current density, inducing premature ventricular complexes and polymorphic ventricular tachycardia.^50^ Notably, the male predominance in Brugada syndrome suggests sex-specific KCNE5 regulation, although the underlying mechanisms, such as hormone-dependent expression, require further elucidation.^51^

### Brain

**Figure 1:**
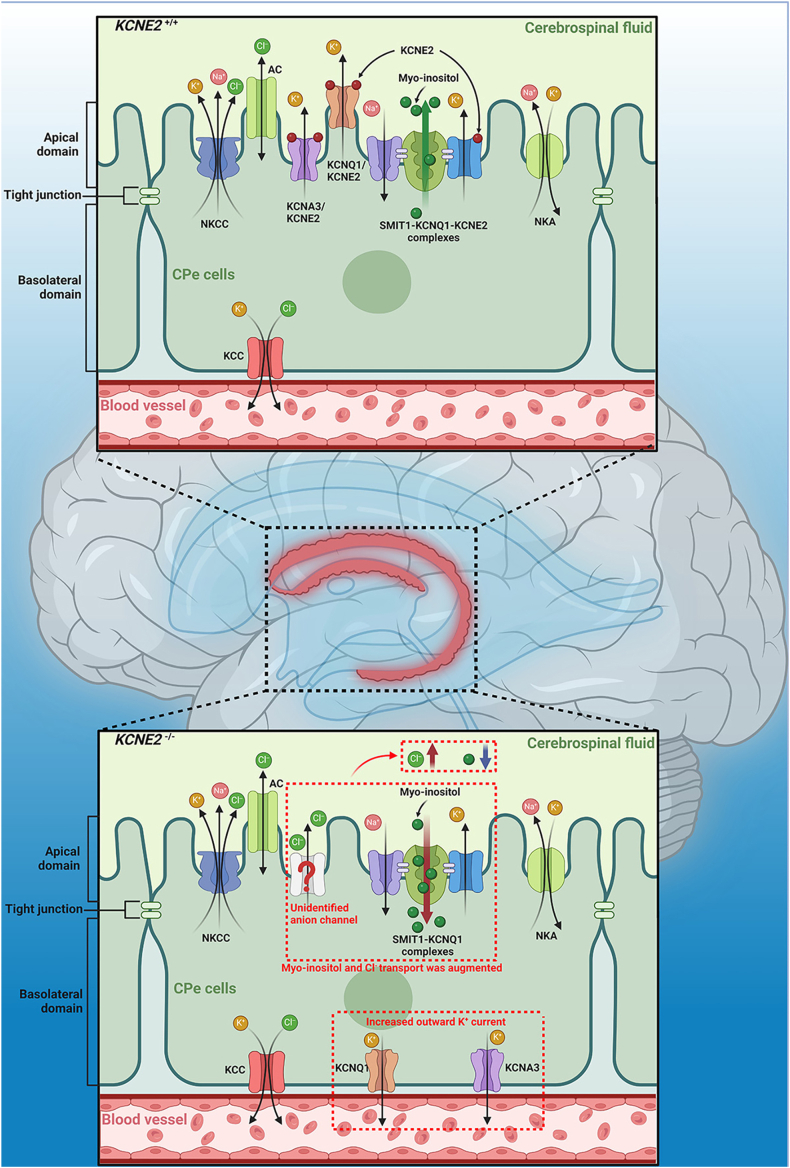
Schematic diagram of ion transport pathways in cerebrospinal fluid epithelial cells. KCNE2 can form a complex with KCNQ1 and KCNA3, which is responsible for transferring potassium ions into the cerebrospinal fluid. Additionally, KCNE2 can also form a complex with KCNQ and SMIT1, preventing SMIT1 from transferring inositol from the cerebrospinal fluid into the cell. When the KCNE2 gene is knocked out, there is abnormal transport of potassium ions from inside the cell to the blood, accompanied by an abnormal accumulation of chloride ions in the cerebrospinal fluid. The mechanism underlying which ion channels are involved in chloride ion transport remains unclear. Furthermore, with the knockout of the KCNE2 gene, there is a significant decrease in the inositol concentration in the cerebrospinal fluid, leading to increased susceptibility to epilepsy.

The ionic homeostasis of cerebrospinal fluid is fundamental for maintaining neuronal function and signal transmission, a process that relies on the coordinated activity of ion channels and transporters in choroid plexus epithelial cells.^52^ KCNE2 plays a pivotal role in central nervous system ion homeostasis by regulating K^+^ and Cl^−^ transport networks in choroid plexus epithelial cells. The K_v_7.1–MiRP1 and K_v_1.3–MiRP1 complexes mediate K^+^ efflux and Cl^−^ secretion into the cerebrospinal fluid, and their functional impairment (*e.g.*, I57T/Q9E mutations) disrupts the cerebrospinal fluid ionic balance—aberrant K^+^ efflux and dysregulated Cl^−^ secretion collectively destabilize the neuronal microenvironment.^53^^,^^54^ Animal studies have revealed that KCNE2 knockout mice exhibit a 50% reduction in the cerebrospinal fluid concentration of myo-inositol, a critical osmolyte that balances osmotic gradients between the cerebrospinal fluid and the neuronal cytosol to prevent cellular edema and maintain electrochemical signaling fidelity.^55^ This imbalance impairs neurocellular volume regulation, leading to neuronal hyperexcitability.^56^ Concurrently, these mice demonstrated increased susceptibility to seizures. While exogenous myo-inositol supplementation partially rescues epileptic phenotypes, the membrane abundance of the sodium-coupled myo-inositol transporter SMIT1 remains unaffected, suggesting that KCNE2 maintains neuroexcitatory equilibrium through mechanisms independent of classical transport pathways, such as the modulation of neuronal membrane potentials or synaptic ion gradients (Fig. 1).^57^ These findings elucidate the potential mechanisms underlying neurological comorbidities in long QT syndrome patients harboring KCNE2 mutations. In addition, changes in MiRP2 expression can similarly affect the gating and function of potassium channels, including those in the K_v_2.1 and K_v_3.1b families in the mammalian brain. These findings suggest that KCNE3 may help maintain the balance of ionic currents in the central nervous system by regulating the function of multiple delayed rectifier potassium currents, which is essential for proper neuronal function and helps prevent pathophysiological conditions, such as hyperexcitability and seizure susceptibility.^58^

Currently, research on the regulation of the nervous system by the KCNE family is still very limited. Preliminary studies suggest that the KCNE family may influence neuronal excitability and signaling by modulating voltage-dependent potassium channel functions,^53^ necessitating further investigation into their roles in the development of neurological disorders, such as epilepsy, depression, and anxiety.^59^^,^^60^ Additionally, the role of KCNE in congenital neurological disorders deserves further exploration in human populations. It would be worth investigating whether patients with LQTS might have potential neurological disorders caused by KCNE gene mutations and whether the KCNE2 gene is related to channel disruptions in familial neonatal epilepsy involving K_v_7.2 or K_v_7.3 on the basis of our existing research foundation.^61^^,^^62^

### Kidney

The KCNE family plays multifaceted roles in renal ionic homeostasis through isoform-specific regulation of potassium channels. In the proximal tubule, MinK colocalizes with K_v_7.1 to facilitate K^+^ delivery to distal nephron segments.^63^ KCNE1 knockout mice exhibit impaired proximal tubular K^+^ flux, leading to polyuria and increased urinary excretion of Na^+^, Cl^–^, and glucose due to defective membrane repolarization during Na^+^-coupled transport.^64^ While overall K^+^ excretion remains unchanged via compensatory secretion in distal segments, these mice develop chronic hypokalemia and hyperaldosteronism-like phenotypes characterized by elevated plasma aldosterone and renin levels.^64^^,^^65^ Notably, dietary manipulations (low-K^+^ or high-K^+^ diets) reversibly modulate aldosterone secretion without affecting adrenal synthesis, suggesting the control of aldosterone production by extracellular K^+^ rather than adrenal dysfunction.^66^ This electrolyte imbalance parallels findings in KCNE3 knockout mice, which similarly exhibit aldosterone elevation but distinct adrenal lymphocytic infiltration. However, the model shows neither adrenal hyperplasia nor neoplasia, implicating divergent molecular pathways in KCNE-mediated aldosterone regulation.^67^

The function of MinK extends beyond its common role in potassium channel regulation, encompassing debated interactions with anoctamin channels. Initial reports posited that MinK co-assembles with TMEM16A chloride channels, converting their gating mechanism from calcium-dependent to voltage-dependent activation and potentially contributing to inherited pathologies.^20^ However, a follow-up study in renal proximal tubules, where TMEM16A mediates critical electrolyte transport, revealed no endogenous KCNE1 expression, with angiotensin II and ATP activating TMEM16A independently of MinK. When heterologously co-expressed, MinK failed to reconfigure TMEM16A into a voltage-gated channel or disrupt its calcium sensitivity, challenging the proposed functional partnership.^68^ This tissue-specific contradiction underscores that MinK’s regulatory roles require contextual validation within physiological microenvironments, necessitating future studies to resolve the molecular basis of its debated interaction with TMEM16A chloride channels in native tissues where both partners coexist, directly probe whether MinK modulates channel gating kinetics or calcium sensitivity under authentic membrane compositions and signaling contexts, and determine if disease-associated MinK variants disrupt chloride homeostasis through this putative partnership to reconcile conflicting pathological models.

**Figure 2:**
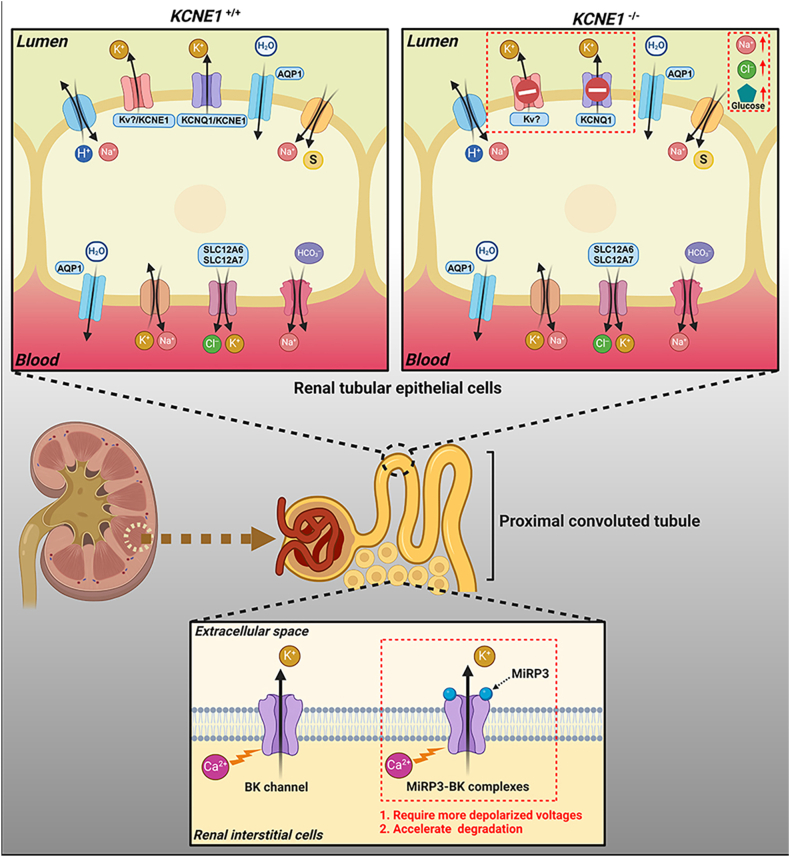
Schematic diagram of KCNE1 regulation in renal proximal tubular epithelial cells. KCNE1 can form a complex with KCNQ1 to secrete potassium ions from inside the cell into the renal tubule lumen. When KCNE1 is knocked out, the loss of sodium ions, chloride ions, and glucose in the urine increases. However, inhibiting the KCNQ1 channel does not significantly disrupt ion secretion, suggesting that KCNE1 may exert its effects by regulating other ion channels.

Transitioning to distal nephron regulation, MiRP3 modulates renal interstitial cells by co-assembling with BK channels (big potassium channels). These channels act as large-conductance Ca^2+^-activated K^+^ channels that hyperpolarize membranes by coupling intracellular Ca^2+^ signals to K^+^ efflux.^69^ MiRP3 increases the activation threshold and reduces the half-life of BK channels, fine-tuning flow-independent K^+^ secretion in a species-specific manner.^70^ In rats, this mechanism prevents hyperkalemia during high-K^+^ intake by enhancing BK-mediated K^+^ efflux while down-regulating secretion under hypokalemic conditions. These findings underscore the adaptive role of KCNE4 in maintaining potassium balance, although its full functional spectrum in renal interstitial cells warrants further exploration (Fig. 2).^70^

### Skeletal muscle

The generation and repolarization of action potentials in skeletal muscle are precisely regulated by voltage-gated ion channels, in which K^+^ channels accelerate repolarization by mediating outward K^+^ fluxes, thereby maintaining a stable resting membrane potential.^71^ MiRP2 forms a functional complex with the voltage-gated potassium channel α-subunit K_v_3.4, which jointly regulates resting membrane potential and muscle excitability. In KCNE3-knockout mice, the sustained outward potassium current density is reduced, the decay of transient outward currents is accelerated, and the transcription and protein expression levels of K_v_3.4 are decreased.^71^^,^^72^ Additionally, the KCNE3–R83H mutation diminishes outward potassium flux, disrupting native skeletal muscle channel function and altering resting membrane potential.^71^ Despite robust evidence from murine models supporting KCNE3’s regulatory role in skeletal muscle electrophysiology, human genetic studies indicate limited pathological relevance.^73^ The frequency of the R83H mutation in patients with periodic paralysis does not significantly differ from that in healthy individuals, and most mutation carriers do not exhibit muscle weakness symptoms.^74^ This discrepancy may arise from individual differences in modifier genes or region-specific expression of the mutation in skeletal muscle. Thus, whether KCNE3 mutations directly cause human skeletal muscle dysfunction remains controversial.

### Smooth muscle

Potassium channels critically govern vascular smooth muscle excitability by modulating membrane potential, and hyperpolarization reduces voltage-gated Ca^2+^ influx, promoting vasodilation, whereas impaired K^+^ efflux induces depolarization-enhanced vasoconstriction.^75^ Within the pulmonary vasculature, KCNE4 is constitutively expressed across the artery. Its knockdown reduces K_v_7.4 channel membrane abundance, induces membrane depolarization, and enhances vasoconstrictor responses, concomitant with decreased total K_v_7.4 protein levels.^76^ Notably, KCNE4 is essential for the electrophysiological effects and vasodilatory actions of classic K_v_7 activators such as retigabine, whereas URO-K10-induced pulmonary vasodilation remains unaffected by KCNE4 knockdown. These findings imply that KCNE4 modulates therapeutic responses to PAH-targeted drugs by regulating K_v_7.4 membrane trafficking, underscoring its potential as a precision therapeutic target.^77^

Extending to cerebrovascular regulation, KCNE subunits similarly govern vascular tone. Cerebral arterial smooth muscle relies on K_v_7.4 and K_v_7.5 complexes requiring auxiliary subunits for stability. Here, MiRP3 enhances K_v_7.4 membrane localization and left-shifts voltage-dependent activation under dihydrotestosterone regulation. Compared with males, females exhibit compensatory doubling of K_v_7.4 expression, suggesting heightened male vulnerability to cerebrovascular dysfunction upon KCNE4 deficiency. Moreover, cerebral KCNE5 dysregulation correlates with neurodegeneration. Tauopathy models show reduced arterial MiRP4 alongside K_v_7.3, K_v_7.5, and K_v_2.1 down-regulation, impairing K_v_7-mediated vasodilation. This deficit likely exacerbates Alzheimer’s pathology by compromising cerebral perfusion, positioning KCNE regulatory networks as therapeutic targets for neurovascular disorders. Further studies confirmed that vascular K_v_7 channels are key effectors of receptor-mediated vasodilation and that their dysfunction disrupts neurovascular coupling, highlighting KCNE subunit regulatory networks as novel therapeutic targets for improving cerebral blood flow disorders.

### Auditory system

The normal function of potassium channels is essential for maintaining ionic homeostasis in the inner ear microenvironment, where they precisely regulate potassium recycling to establish the electrochemical foundation for auditory transduction.^78^ In this process, the KCNE1 channel plays a central role and is specifically expressed in marginal cells of the cochlear duct and vestibular dark cells. It mediates the active transport of high-concentration K^+^ into the extracellular fluid, establishing the unique high-K^+^, low-Na^+^ environment of the endolymph. This distinct ionic gradient not only underpins mechanoelectrical transduction in hair cells but also provides the requisite electrical driving force for auditory signal generation and propagation. Genetic deletion of KCNE1 disrupts the endolymphatic K^+^ concentration, volume, and pressure, thereby impairing hair cell and auditory neuronal function. Notably, such K^+^ dysregulation often coexists with cardiac conduction abnormalities in Jervell and Lange–Nielsen syndrome or noise-induced hearing loss, underscoring the multisystemic importance of potassium channels.^79^^,^^80^ The presence of MinK subunits can partially rescue loss-of-function mutations in heterozygous individuals, offering a molecular explanation for certain long QT syndrome cases without hearing impairment.^81^ Additionally, deficiency of lysosome-associated membrane glycoprotein 2 (LIMP2) indirectly disrupts K^+^ homeostasis by destabilizing the K_v_7.1–MinK complex, ultimately leading to hearing loss.^82^ Recent studies demonstrate that adeno-associated virus serotype 1 (AAV1)-mediated gene delivery via posterior semicircular canal injection effectively rescues hearing loss in KCNE1 knockout mice. The viral construct employs a hybrid CB7 promoter comprising cytomegalovirus (CMV) enhancer and chicken β-actin (CAG) core elements to drive strong, cell-specific KCNE1 transgene expression in cochlear marginal cells of the stria vascularis, with high-dose treatment in neonatal mice sustaining improved auditory and vestibular function for at least 5 months, although long-term efficacy and safety require further validation.^83^ Beyond KCNE1, KCNE3 has been implicated in auditory processing through modulation of K_v_4.2 channel activity in spiral ganglion neurons, the bipolar primary afferent neurons that relay mechanoelectrical signals from cochlear hair cells to the cochlear nucleus in the brainstem.^84^^,^^85^ Its deletion abolishes resting membrane potential variability and induces age-dependent alterations in action potentials, further corroborating the critical role of potassium channel regulation in auditory pathways.^84^

### Digestive system

In pancreatic duct cells, multiple potassium channels contribute to anion secretion by maintaining the electrochemical driving force, and their functional enhancement may offer therapeutic strategies to restore impaired HCO3^–^ and fluid secretion in conditions such as pancreatitis.^86^ Studies have demonstrated that K_v_7.1 co-assembles with MinK in murine pancreatic acinar cells and closely resembles those of cardiac K_v_7.1–MinK complexes. KCNE1 knockout markedly attenuates K_v_7.1 currents and disrupts channel membrane trafficking in the exocrine pancreas, indicating that KCNE1 is essential for KCNQ1 functional expression.^87^ Dysregulation of this mechanism may compromise pancreatic secretory homeostasis, providing a molecular basis for exploring KCNE1-targeted interventions.

Gastric parietal cells secrete H^+^ into the lumen via apical H^+^–K^+^–ATPase while concurrently absorbing K^+^.^12^ Apical K_v_7.1–MiRP1 potassium channels sustain gastric acid secretion by replenishing luminal K^+^. As a regulatory subunit, MiRP1 converts acid-inhibited K_v_7.1 into an acid-activated complex through its extracellular NH_2_-terminus and transmembrane domains, increasing channel activity in acidic environments. The continuous supply of luminal K^+^ by K_v_7.1–MiRP1 provides the electrochemical driving force for H^+^ secretion.^88^ KCNE2 knockout mice exhibit severe achlorhydria, parietal cell atrophy, gastric hyperplasia, and hypergastrinemia, with hyperplasia arising from increased nonacid-secreting cell populations. Chronic omeprazole (proton pump inhibitor) treatment in wild-type mice induces shifts in the gut microbiome β diversity, which converges toward KCNE2 knockout-like microbiota. Notably, KCNE2 deficiency reduces Bacteroidales abundance (positively correlated with human cardiovascular risk) via suppression of acid secretion.^89^

Unlike the gastric function of KCNE2, KCNE3 knockout does not induce gastric morphological defects, but its basolateral localization in glands suggests roles in luminal salt, enzyme, and acid secretion.^90^^,^^91^ In KCNE2/KCNE3 double knockouts, K_v_7.1 retains its apical localization, yet KCNE3 deficiency exacerbates gastric abnormalities, whereas KCNE2 loss triggers compensatory KCNE3 up-regulation and K_v_7.1 mislocalization to the basolateral membrane.^92^ Widely co-expressed in the small and large intestines, the K_v_7.1–MiRP2 complex mirrors the crypt–villus distribution of cystic fibrosis transmembrane conductance regulators. Functioning as constitutively open channels with linear voltage dependence, they form the molecular basis for colonic Cl^−^ secretion. cAMP signaling activates these channels via basolateral K^+^ recycling and electrochemical gradients. While cystic fibrosis attenuates this pathway, secretory diarrhea, such as that caused by cholera, hyperactivates it.^91^ Intriguingly, intestinal epithelia retain residual anion secretion upon K_v_7.1–MiRP2 loss, which is primarily compensated by TASK-2 two-pore K^+^ channels.^93^ Taken together, these data indicate that estrogen regulates the expression of K_v_7.1 and MiRP2 and affects Cl^–^ secretion by regulating the gating and pharmacological properties of these ion channels, and that dysfunction of K_v_7.1–MiRP2 may partly result in ulcerative colitis and colon cancer.^94^, ^95^, ^96^

### Lung

The transepithelial secretion of fluids, mucins, and electrolytes by airway epithelial cells is critical for airway surface liquid homeostasis, a process dependent on the coordinated activation of apical Cl^−^ channels and basolateral K^+^ channels.^97^ Cl^−^ channels mediate Cl^−^ efflux across the apical membrane, whereas basolateral K^+^ channels sustain membrane hyperpolarization through K^+^ recycling, thereby driving transepithelial electrolyte transport.^98^ In cystic fibrosis, the pathological hallmark is defective cAMP-dependent Cl^−^ conductance at the apical membrane of airway epithelia; however, Cl^−^ secretory function also relies on parallel activation of basolateral K^+^ channels to maintain electrochemical gradients.^99^ Studies indicate that the K_v_7.1–MiRP2 complex in murine tracheal epithelia likely constitutes basolateral K^+^ channels and that its functional impairment disrupts cAMP-dependent regulation of Cl^−^ secretion, suggesting that MiRP2 indirectly modulates Cl^−^ transport by sustaining K^+^ channel activity. This mechanism provides new insights into ionic transport defects in cystic fibrosis and identifies the K_v_7.1–MiRP2 complex as a potential therapeutic target. Additionally, KCNE2 and KCNQ1 form channel complexes in the lungs, and KCNE2 deficiency is associated with reduced KCNQ1 expression and diminished resistance to lung ischemia‒reperfusion injury, highlighting KCNE2’s role in pulmonary homeostasis in mice.^100^

### Endocrine system

Extensive knockout of the KCNE2 gene results in a series of multisystem syndromes, disrupting the systemic endocrine system and causing endocrine disorders, such as diabetes, hypercholesterolemia, hyperkalemia, anemia, and elevated angiotensin II.^101^ Specifically, targeted disruption of KCNE2 impairs thyroid iodide accumulation, halves the T4 content in breast milk, and leads to hypothyroidism, a 50% reduction in offspring number, dwarfism, alopecia, goiter, and myocardial dysfunction, including hypertrophy, fibrosis, and a reduced shortening fraction.^102^ In addition to cardiac electrophysiological regulation, KCNE family members exert systemic cardiovascular effects due to KCNE proteins in different hormone-producing tissues in animal models. KCNE2 deletion leads to a series of extracardiac damage events that can, in turn, affect the heart. KCNE2 deficiency results in hypothyroidism, which alters cardiac structure and impairs contractility, whereas mistransport of K_v_7.1 in parietal cells of the intestine results in the transfer of K^+^ into the bloodstream, causing arrhythmogenic hyperkalemia.^101^^,^^102^ The use of the proton pump inhibitor omeprazole to inhibit gastric acid secretion can extend the lifespan of heart-specific KCNE2 knockout mice.^89^ Notably, KCNE2 loss paradoxically reduces infarct size during acute myocardial ischemia while promoting atherosclerosis and diet-dependent electrical remodeling to increase the risk of long-term sudden death, highlighting the dualistic nature of its pathophysiological effects.^103^^,^^104^ In contrast with KCNE2’s metabolic‒cardiac interplay, KCNE3 may modulate cardiac electrical activity through an adrenal–cardiac axis. KCNE3 knockout mice develop secondary hyperaldosteronism with adrenal lymphocytic infiltration, triggering aldosterone-dependent QT prolongation and atrial/ventricular fibrillation.^67^^,^^105^ This finding suggests potential regulation through the renin‒angiotensin‒aldosterone system and warrants investigation into its role in fluid homeostasis and the hematological system. Moreover, KCNE2 is essential for normal pancreatic β-cell electrical activity and insulin secretion, as inhibiting K_v_7.1 can increase glucose-stimulated insulin secretion in these cells.^106^, ^107^, ^108^ Although the underlying mechanisms of these diseases are not fully understood, no other member of the KCNE gene family has been found to regulate the body as extensively as KCNE2 does. The precise physiological roles and molecular pathogenesis of related disease states make KCNE2 a significant target for future research.

Sex steroids critically modulate KCNE-mediated electrophysiological functions across tissues in animal models, as evidenced by divergent hormonal pathways employed by KCNE2 and KCNE4 to establish cardiac electrophysiological sex differences. KCNE2 expression in mice is specifically up-regulated by 17-β estradiol during late pregnancy, implying protective roles in gestational cardiac adaptation.^109^ Conversely, KCNE4 is strongly androgen-dependent; its expression in male hearts significantly exceeds that in female hearts, with 5α-dihydrotestosterone directly inducing transcription.^39^ Although MiRP3 was reported to inhibit K_v_7.1 in heterologous systems,^5^ elevated cardiac KCNE4 expression in males correlates with enhanced repolarizing current density through augmentation of both the transient outward potassium current (Ito) and ultrarapid delayed rectifier current (IKur), generated by K_v_4.2 and K_v_1.5 subunits, respectively, paralleling the functional roles of KCNE2 in murine ventricles.^39^^,^^110^^,^^111^ Such polarized hormonal regulation becomes clinically relevant under pathological conditions: KCNE2 deficiency may exacerbate arrhythmia risk during estrogen fluctuations, whereas KCNE4 deletion could eliminate androgen-mediated K_v_ regulation, causing marked repolarization impairment and age-dependent ventricular arrhythmia susceptibility in males.^39^^,^^109^ This tissue-specific hormonal modulation extends to the colon, where estrogen critically regulates the K_v_7.1–MiRP2 channel complex. This complex has been shown to mediate cAMP-activated Cl^−^ secretion by recycling K^+^.^112^ In females, K_v_7.1–MiRP2 expression fluctuates with the estrogen cycle, and 17-β estradiol rapidly dissociates K_v_7.1 and MiRP2 subunits, reducing channel currents to form an anti-secretory effect. Male intestinal epithelia exhibit higher MiRP2 expression and greater sensitivity to Kv7.1 inhibitors. A phosphorylation-sensitive mutation in KCNE3 (S82A) alters estrogen responsiveness, confirming direct hormonal control of channel function.^94^ Dysregulation of this pathway may contribute to sex-biased pathologies, including ulcerative colitis and secretory diarrheas.

## Future perspective

Since the discovery of almost all KCNE isoforms, increasing research has spurred interest in the investigation of the KCNE gene family. As modulators of various ion channels, KCNE genes have become particularly important in the pathogenesis of various cardiac arrhythmias. Early studies focused primarily on their roles in heart and hereditary arrhythmia syndromes. The recent identification of KCNE6 in zebrafish, a novel KCNE member that co-assembles with KCNQ1 to generate slow-activating IK-like currents despite its sequence homology to KCNE3, highlights evolutionary divergence in channel modulation. In addition to their regulatory roles in the heart, extrinsic cardiac regulation by KCNEs can also induce significant arrhythmias, making it valuable to study the regulation of KCNEs on the heart and arrhythmias from a broader perspective.^66^ Furthermore, arrhythmias induced by mutations and structural changes in KCNE genes are often associated with these genes. For example, variations, such as long and short KCNE3 and KCNE4 variants, are known to influence the induction of arrhythmias, which warrants further investigation in the future.^113^

With the in-depth study of the functional roles of the KCNE gene family, the potential of KCNE in disease diagnosis and treatment is gradually becoming apparent (Table 2). KCNE plays a crucial role not only in the regulation of cardiac electrical activity but also in its widespread distribution in various tissues, including blood vessels and tumors. No KCNE-targeting drugs have reached clinical use to date, highlighting a critical gap between mechanistic understanding and therapeutic translation. Future research is expected to uncover deeper mechanisms of KCNE in cardiovascular diseases, cancers, and neurological disorders, paving the way for its development as a disease biomarker or therapeutic target. For example, the specific expression of KCNE3 in angiogenesis offers a new avenue for the diagnosis of vascular diseases,^114^ whereas the up-regulation of KCNE4 in colorectal cancer suggests its critical role in the tumor microenvironment and immune evasion, potentially providing new breakthroughs for cancer treatment.^115^ Despite promising preclinical compounds that modulate interactions of KCNE and K_v_ at the cellular level, their therapeutic efficacy and specificity *in vivo* remain unproven. Additionally, with the discovery of various regulatory molecules, the prospects for drug development targeting KCNE regulatory mechanisms are promising. These studies may not only lead to precise treatment strategies based on ion channels but also provide more effective solutions for improving patient prognosis and reducing drug side effects. Therefore, the application prospects of KCNE in disease diagnosis and personalized therapy warrant further exploration. Various compounds have been investigated at the cellular level to validate the potential regulatory value of KCNE as a therapeutic target for potassium ion channel modulation. These compounds act through specific mechanisms involving interactions between KCNE subfamily members and Kv, thereby influencing the electrophysiological properties of cells. The further development of these compounds not only has the potential to improve ion channel function but also may offer therapeutic prospects for reversing electrophysiological abnormalities caused by genetic mutations.

**Table 2:** Effects of KCNE-related diseases on different organs and tissues.

Organs/tissues	Disease	KCNE	Effects of KCNE	References
Heart	LQTS	KCNE1	Decreased IKs currents caused by mutations of KCNE1–S74L, D76N, leading to delayed cardiac repolarization	120,134
			Presented the genetic test results of individuals’ risk for LQTS due to the KCNE1 mutation in patients	13,79,135, 136, 137, 138
			Presented familial long QT syndrome in the Japanese patients caused by mutations of KCNE1–S38G, D85N	139
			Decreased concentration of K^+^ and increased aldosterone secretion in a KCNE1-knockout mouse model, enhancing the risk of ventricular arrhythmia	66
			Reduced IKs currents amplitude by 50 % caused by mutation of KCNE1–G52R, leading to delayed cardiac repolarization	119
			Presented prolonged epicardial and endocardial APD90 (action potential duration at 90 % repolarization), frequent epicardial early afterdepolarizations, spontaneous ventricular tachycardia, and action potential alternans by KCNE1 knockout	37
			Down-regulated KCNQ1/KCNE1-D76N complex leading to decreased IKs currents	140
			Disrupted normal assembly of KCNQ1/KCNE1 complex caused by mutation of KCNE1–T58P/L59P	141
			Prolonged QT interval extremely and resulted in torsade de pointes after administration of sodium channel blockers in a child patient undergoing Fontan procedure with the KCNE–D85N mutation	142
			Prolonged QT interval significantly and caused torsade de pointes in a 72-year-old woman carrying the KCNE1–G38S mutation with unsolved hypokalemia and hypomagnesemia	124
			Presented the relation between KCNE–D85N and drug-induced long QT syndrome	143
		KCNE2	Enhancing the risk of drug-induced long QT syndrome caused by allelic variants of KCNE2	33
			Decreased Ikr currents leading to auditory stimulus-induced arrhythmia under the condition of hypomagnesaemia and hypocalcemia caused by the KCNE2–T10M mutation	34
			Decreased IKs by 50 % and prolonged ventricular action potential duration	110
			Manifested that additional stressors were required to induce a clinical phenotype of LQTS with functional loss of KCNE2	144
		KCNE3	Presented genetic test results of individuals risk for LQTS by mutation of KCNE3 in patients	145
			Shorten QT interval and accelerated cardiac repolarization by the ectopic expression of E3 in cardiac myocytes	40
			Delayed ventricular repolarization in an aldosterone-dependent manner, leading to extracardiac arrhythmogenesis	67
	Atrial fibrillation	KCNE1	Presented the genetic test results of individuals’ risk for atrial fibrillation due to the KCNE1 mutation in patients and populations	133,146, 147, 148, 149, 150, 151, 152, 153
			Increased outward current and shortened atrial action potentials in atrial myocytes by KCNE1 knockout	154
			Prolonged the atrial action potential and reduced the frequency for alternans behavior by KCNQ1/KCNE1–38G	128
			Enhanced potential risk of lone atrial fibrillation in patients with KCNE1–G38S mutation	125,155, 156, 157
			Enhanced potential risk of new-onset postoperative atrial fibrillation after lung lobectomy with the decreased expression of KCNE1	158
			Revealed the relation between postoperative atrial fibrillation and 112G > A polymorphism of KCNE1	159
			Enhanced IKs currents and expression of KCNE1 and decreased ICaL in chronic atrial fibrillation patients due to the expression of Pitx2c	160
		KCNE2	Presented genetic test results of individuals’ risk for atrial fibrillation due to the KCNE2 mutation in patients and populations	131,152,161,162
		KCNE3	Enhanced activity of K_v_4.3/KCNE3 and K_v_11.1/KCNE3 causing faster cardiac action potential repolarization by KCNE3–V17M mutation	163
		KCNE5	Enhanced IKs currents of KCNQ1/KCNE1/KCNE5–L65F	48
	Brugada syndrome	KCNE2	Increased Ito current density and slowed the inactivation rate by KCNE2–M54T, I57T	164
		KCNE3	Increased Ito intensity of KCND3/KCNE3–R99H and KCND3/KChIP2b/KCNE3–T4A	165
			Exhibited a Brugada-pattern electrocardiogram in a patient with KCNE3–T4A mutation	166
		KCNE5	Increased Ito currents of KCND3/KCNE5–D92E, E93X	47
			Augmented currents mediated by K_V_1.5 and K_V_2.1 channels, resulting in increased ventricular current density and enhanced susceptibility to arrhythmias	50
	Heart failure	KCNE1	Presented the association between KCNE1–S38G and heart failure in two populations	167
		KCNE2	Delayed progress of terminal heart failure using the proton-pump inhibitor in a cardiac-specific KCNE2-knockout strain	89
	Sudden cardiac death	KCNE2	Generated a multisystem syndrome including diabetes mellitus, hypercholesterolemia, hyperkalemia, anemia, atherosclerosis, and elevated angiotensin II in KCNE2-knockout mice, causing a predisposition to sudden cardiac death	101,104
			Attenuated acute heart ischemia/reperfusion injury by KCNE2 knockout	103
Pancreatic β cells	Type 2 diabetes mellitus	KCNE2	Disrupted the glucose tolerance in a Western diet and insulin secretion, reduced the expression of insulin receptor in skeletal muscle tissue, and decreased the β-cell peak outward K^+^ current by KCNE2 knockout	107
Skeletal muscle	Periodic paralysis	KCNE3	Resulted in less outward current and a diminished capacity to set resting potential in the K_v_3.4/KCNE3–R83H complex, compared with the wild-type, leading to the dysfunction of the muscle	71,168
			Resulted in abnormal development of skeletal muscle and loss of the typical biphasic decline in contractile force with decreased expression of KCNC4 and KCNH2 and increased expression of KCNK4 in the gastrocnemius of KCNE3-knockout mice	72
			Resulted in enhancing susceptibility to thyrotoxic hypokalemic periodic paralysis in one sporadic case of KCNE–R83H	169
			Revealed that no significant difference in KCNE3–R83H between periodic paralysis patients and the healthy population was found	73
Thyroid	Hypothyroidism	KCNE2	A multiple syndrome including hypothyroidism, dwarfism, alopecia, goiter, and cardiac abnormalities, mainly caused by abnormal thyroid iodide accumulation in KCNE2-knockout mice	102,170
Stomach	Achlorhydria	KCNE2	Disrupted the proton secretion, parietal cell morphology, gastric glandular, and KCNQ1 distribution, leading to achlorhydria and hypergastrinemia in KCNE2-knockout mice	88
	Gastric cancer	KCNE2	Exhibited a gastric preneoplastic phenotype, including gastritis cystica profunda, increased stomach mass, increased expression of Ki67 and nuclear Cyclin D1 expression, and TFF2- and cytokeratin 7-expressing metaplasia	10
			Down-regulated the expression of KCNE2 significantly in gastric cancer tissues	171
	Achlorhydria-induced iron-deficient anemia	KCNE2	Decreased plasma iron with KCNE2-knockout and exhibited developed anemia only in males	172
Colon	Colorectal cancer	KCNE4	Promoted tumor-promoting phenotypes in cancer progression by up-regulating the expression of KCNE4 and contributed to the radio resistance through the PI3K/AKT signaling pathway	115,173
Liver	Nonalcoholic fatty liver disease	KCNE2	Resulted in nonalcoholic fatty liver disease mainly through iron deficiency, which could be reversed by iron supplementation in KCNE2-knockout mice	174
	Hepatic ischemia‒reperfusion injury	KCNE4	Exacerbated liver damage in aged male mice in the context of ischemia‒reperfusion injury, influenced by hormonal and gender-specific factors, by KCNE4 knockout	175
Lung	Pulmonary ischemia‒reperfusion injury	KCNE2	Decreased expression of KCNQ1 and KCNB1 and resistance to pulmonary ischemia/reperfusion injury	100
	Pulmonary arterial hypertension	KCNE4	Enhanced activity of the KCNQ1 channel with the up-regulation of KCNE4	176
Brain	Alzheimer’s disease	KCNE3	Inhibited Abeta peptide-mediated cell death by suppressing K_v_3.4/KCNE3	177
		KCNE5	Decreased KCNE5 expression in tau transgenic mice, with a decrease in the expression of K_v_7.3, K_v_7.5, and K_v_2.1	178
	Seizure	KCNE2	Decreased myo-inositol concentration in cerebrospinal fluid, resulting in higher stress and seizure susceptibility in KCNE2-knockout mice	57
Ear	Meniere’s disease	KCNE1/KCNE3	Increased susceptibility to Meniere’s disease by variants of KCNE1 and KCNE3, but with no association in caucasians	179, 180, 181
Lymph nodes	Metastatic melanoma	KCNE4	Promoted the metastasis of the metastatic murine melanoma cell line from the tongue to the lymph node with upregulated expression of KCNE4	182
Testis	Testis atrophy	KCNE1	Disrupted the process of germ-cell development through KCNEQ1/KCNE1 due to the lack of expression of KCNQ1 and KCNE1	183

In-depth exploration of KCNE’s role in interorgan interactions and the upstream and downstream regulatory mechanisms of KCNE gene expression is lacking. The expression of the KCNE gene family not only plays a crucial role in individual organs but also coordinates regulation between different organs through complex signaling pathways and molecular networks. The interaction between the heart and gastrointestinal tract may influence cardiac electrical activity and gastrointestinal function via KCNE-regulated ion channels. Understanding these cross-organ interactions will help reveal the pathological mechanisms underlying various systemic diseases. Furthermore, identifying and analyzing the upstream signaling molecules and downstream effectors that regulate KCNE gene expression, such as transcription factors, microRNAs, and protein kinases, could lead to comprehensive therapeutic strategies for complex diseases such as cancer. Therefore, systematic research on the regulation of KCNE gene expression and its interactions across different organs and tissues holds great potential.

## Conclusion

**Figure 3:**
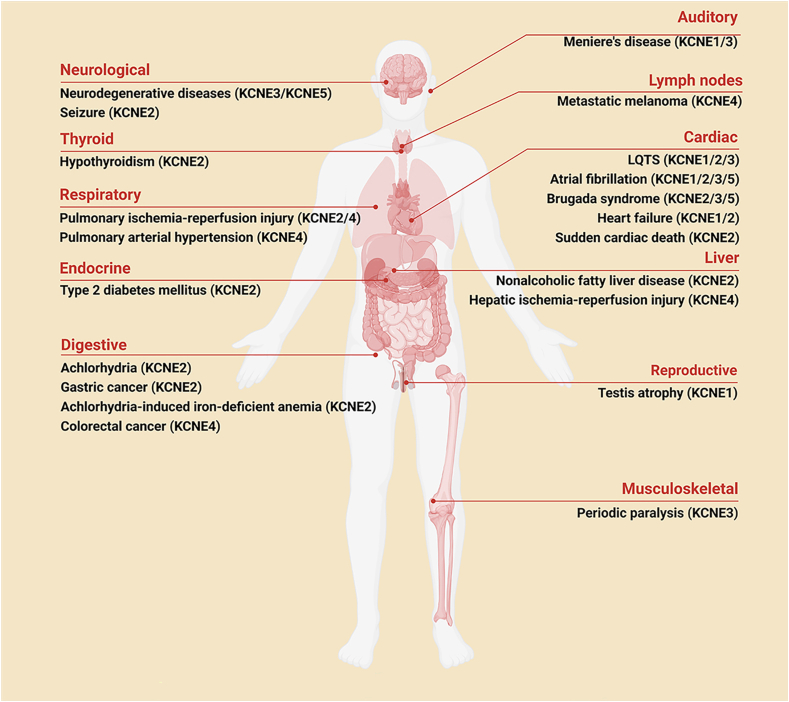
Knocking out different KCNE genes may lead to multisystem or tissue diseases throughout the body. Knockout of the KCNE gene may cause cardiac conduction abnormalities and congenital long QT syndrome, whereas deletion of the KCNE2 gene may result in abnormal gastric acid secretion and associated gastrointestinal issues. Additionally, the knockout of other KCNE genes could be linked to respiratory dysfunction or neurological disorders. As shown in the figure, the wide-ranging impact of these gene knockouts on multiple systems underscores the critical role of KCNE genes in maintaining normal physiological function.

In summary, as β-subunits regulate ion channels, the primary function of the KCNE gene family is to regulate K_v_. Members of this gene family have been confirmed to play significant regulatory roles in various organs and tissues, including the heart, gastrointestinal tract, thyroid, choroid plexus, and vascular endothelium. By influencing K_v_ channels in these tissues, the KCNE gene family plays crucial roles in both physiological and pathological processes (Fig. 3).

Owing to the different roles that KCNE gene family members play in various K_v_ channels, they present significant challenges as drug targets. Each KCNE subunit interacts with a specific K_v_, altering the electrophysiological properties and drug sensitivity of the channel. Thus, developing drugs that target KCNE requires a precise understanding of the specific mechanisms of each subunit and their expression and function in different tissues. Although we have gained some understanding of the functions of the KCNE gene family, our current knowledge remains limited. Through further research, we hope to develop more effective therapeutic strategies to address various diseases caused by abnormalities in KCNE genes.

## CRediT authorship contribution statement

**Junshen Xiao:** Writing – review & editing, Writing – original draft, Visualization, Validation, Investigation, Formal analysis, Data curation. **Xu Cheng:** Writing – review & editing, Validation, Investigation, Data curation. **Dou Huang:** Writing – review & editing, Validation, Investigation, Data curation. **Shichao Wei:** Writing – review & editing, Visualization, Validation, Investigation, Data curation. **Zhaoyang Hu:** Writing – review & editing, Supervision, Funding acquisition, Conceptualization.

## Funding

This study was supported by the 10.13039/501100001809National Natural Science Foundation of China (No. 82270326 to Z.H.).

## Conflict of interests

The authors declared no competing interests.

## References

[1] Abbott G.W. (2006). Molecular mechanisms of cardiac voltage-gated potassium channelopathies. Curr Pharm Des.

[2] Takumi T., Ohkubo H., Nakanishi S. (1988). Cloning of a membrane protein that induces a slow voltage-gated potassium current. Science.

[3] Murai T., Kakizuka A., Takumi T., Ohkubo H., Nakanishi S. (1989). Molecular cloning and sequence analysis of human genomic DNA encoding a novel membrane protein which exhibits a slowly activating potassium channel activity. Biochem Biophys Res Commun.

[4] Abbott G.W., Sesti F., Splawski I. (1999). MiRP1 forms IKr potassium channels with HERG and is associated with cardiac arrhythmia. Cell.

[5] Grunnet M., Jespersen T., Rasmussen H.B. (2002). KCNE4 is an inhibitory subunit to the KCNQ1 channel. J Physiol.

[6] Piccini M., Vitelli F., Seri M. (1999). *KCNE1*-like gene is deleted in AMME contiguous gene syndrome: identification and characterization of the human and mouse homologs. Genomics.

[7] Kasuya G., Zempo B., Yamamoto Y., Ryu K., Ono F., Nakajo K. (2024). Identification of KCNE6, a new member of the KCNE family of potassium channel auxiliary subunits. Commun Biol.

[8] Barhanin J., Lesage F., Guillemare E., Fink M., Lazdunski M., Romey G. (1996). K(V)LQT1 and lsK (minK) proteins associate to form the I(Ks) cardiac potassium current. Nature.

[9] McCrossan Z.A., Abbott G.W. (2004). The MinK-related peptides. Neuropharmacology.

[10] Roepke T.K., Purtell K., King E.C., La Perle K.M.D., Lerner D.J., Abbott G.W. (2010). Targeted deletion of Kcne2 causes gastritis cystica profunda and gastric neoplasia. PLoS One.

[11] Heron S.E., Hernandez M., Edwards C. (2010). Neonatal seizures and long QT syndrome: a cardiocerebral channelopathy?. Epilepsia.

[12] Heitzmann D., Warth R. (2007). No potassium, no acid: K^+^ channels and gastric acid secretion. Physiology.

[13] Splawski I., Shen J., Timothy K.W. (2000). Spectrum of mutations in long-QT syndrome genes. *KVLQT1* HERG SCN5A KCNE1 and KCNE2. Circulation.

[14] Sesti F., Goldstein S.A. (1998). Single-channel characteristics of wild-type IKs channels and channels formed with two minK mutants that cause long QT syndrome. J Gen Physiol.

[15] Doyle D.A., Morais Cabral J., Pfuetzner R.A. (1998). The structure of the potassium channel: molecular basis of K^+^ conduction and selectivity. Science.

[16] Barro-Soria R., Rebolledo S., Liin S.I. (2014). KCNE1 divides the voltage sensor movement in KCNQ1/KCNE1 channels into two steps. Nat Commun.

[17] Barro-Soria R., Ramentol R., Liin S.I., Perez M.E., Kass R.S., Larsson H.P. (2017). KCNE1 and KCNE3 modulate KCNQ1 channels by affecting different gating transitions. Proc Natl Acad Sci U S A.

[18] Sastre D., Colomer-Molera M., Roig S.R. (2024). Molecular mapping of KCNE4-dependent regulation of Kv1.3. Am J Physiol Cell Physiol.

[19] Barro-Soria R., Perez M.E., Larsson H.P. (2015). KCNE3 acts by promoting voltage sensor activation in KCNQ1. Proc Natl Acad Sci U S A.

[20] Ávalos Prado P., Häfner S., Comoglio Y. (2021). KCNE1 is an auxiliary subunit of two distinct ion channel superfamilies. Cell.

[21] Brandt M.C., Endres-Becker J., Zagidullin N. (2009). Effects of KCNE2 on HCN isoforms: distinct modulation of membrane expression and single channel properties. Am J Physiol Heart Circ Physiol.

[22] Decher N., Bundis F., Vajna R., Steinmeyer K. (2003). KCNE2 modulates current amplitudes and activation kinetics of HCN4: influence of KCNE family members on HCN4 currents. Pflügers Archiv.

[23] Nerbonne J.M., Kass R.S. (2005). Molecular physiology of cardiac repolarization. Physiol Rev.

[24] Wu D.M., Jiang M., Zhang M., Liu X.S., Korolkova Y.V., Tseng G.N. (2006). KCNE2 is colocalized with KCNQ1 and KCNE1 in cardiac myocytes and may function as a negative modulator of I(Ks) current amplitude in the heart. Heart Rhythm.

[25] Sanguinetti M.C., Curran M.E., Zou A. (1996). Coassembly of K(V)LQT1 and minK (IsK) proteins to form cardiac I(Ks) potassium channel. Nature.

[26] Wattanasirichaigoon D., Beggs A.H. (1998). Molecular genetics of long-QT syndrome. Curr Opin Pediatr.

[27] Park K.H., Piron J., Dahimene S. (2005). Impaired KCNQ1-KCNE1 and phosphatidylinositol-4,5-bisphosphate interaction underlies the long QT syndrome. Circ Res.

[28] Terrenoire C., Clancy C.E., Cormier J.W., Sampson K.J., Kass R.S. (2005). Autonomic control of cardiac action potentials: role of potassium channel kinetics in response to sympathetic stimulation. Circ Res.

[29] Kurokawa J., Bankston J.R., Kaihara A., Chen L., Furukawa T., Kass R.S. (2009). KCNE variants reveal a critical role of the beta subunit carboxyl terminus in PKA-dependent regulation of the IKs potassium channel. Channels.

[30] Wilson Z.T., Jiang M., Geng J. (2021). Delayed KCNQ1/KCNE1 assembly on the cell surface helps I(Ks) fulfil its function as a repolarization reserve in the heart. J Physiol.

[31] Dahimène S., Alcoléa S., Naud P. (2006). The N-terminal juxtamembranous domain of KCNQ1 is critical for channel surface expression: implications in the Romano-Ward LQT1 syndrome. Circ Res.

[32] Li Y., Zaydman M.A., Wu D. (2011). KCNE1 enhances phosphatidylinositol 4,5-bisphosphate (PIP2) sensitivity of IKs to modulate channel activity. Proc Natl Acad Sci USA.

[33] Sesti F., Abbott G.W., Wei J. (2000). A common polymorphism associated with antibiotic-induced cardiac arrhythmia. Proc Natl Acad Sci USA.

[34] Gordon E., Panaghie G., Deng L. (2008). A KCNE2 mutation in a patient with cardiac arrhythmia induced by auditory stimuli and serum electrolyte imbalance. Cardiovasc Res.

[35] Abbott G.W. (2015). The KCNE2 K^+^ channel regulatory subunit: ubiquitous influence, complex pathobiology. Gene.

[36] London B. (2001). Cardiac arrhythmias: from (transgenic) mice to men. J Cardiovasc Electrophysiol.

[37] Thomas G., Killeen M.J., Gurung I.S. (2007). Mechanisms of ventricular arrhythmogenesis in mice following targeted disruption of KCNE1 modelling long QT syndrome 5. J Physiol.

[38] Lundby A., Olesen S.P. (2006). KCNE3 is an inhibitory subunit of the Kv4.3 potassium channel. Biochem Biophys Res Commun.

[39] Crump S.M., Hu Z., Kant R., Levy D.I., Goldstein S.A.N., Abbott G.W. (2016). Kcne4 deletion sex- and age-specifically impairs cardiac repolarization in mice. FASEB J.

[40] Mazhari R., Nuss H.B., Armoundas A.A., Winslow R.L., Marbán E. (2002). Ectopic expression of KCNE3 accelerates cardiac repolarization and abbreviates the QT interval. J Clin Investig.

[41] Jiang M., Zhang M., Tang D.G. (2004). KCNE2 protein is expressed in ventricles of different species, and changes in its expression contribute to electrical remodeling in diseased hearts. Circulation.

[42] Chang P.C., Lin S.F., Chu Y. (2019). LCZ696 therapy reduces ventricular tachyarrhythmia inducibility in a myocardial infarction-induced heart failure rat model. Cardiovasc Ther.

[43] Viskin S., Justo D., Halkin A., Zeltser D. (2003). Long QT syndrome caused by noncardiac drugs. Prog Cardiovasc Dis.

[44] Kojima A., Mi X., Fukushima Y., Ding W.G., Omatsu-Kanbe M., Matsuura H. (2021). Elevation of propofol sensitivity of cardiac I(Ks) channel by KCNE1 polymorphism D85N. Br J Pharmacol.

[45] Baldo B.A. (2023). Allergic and other adverse reactions to drugs used in anesthesia and surgery. Anesthesiol Perioper Sci.

[46] Zhu Y., Liu X., Li Y., Yi B. (2024). The applications and prospects of big data in perioperative anesthetic management. Anesthesiol Perioper Sci.

[47] Ohno S., Zankov D.P., Ding W.G. (2011). KCNE5 (KCNE1L) variants are novel modulators of Brugada syndrome and idiopathic ventricular fibrillation. Circ Arrhythm Electrophysiol.

[48] Ravn L.S., Aizawa Y., Pollevick G.D. (2008). Gain of function in IKs secondary to a mutation in KCNE5 associated with atrial fibrillation. Heart Rhythm.

[49] Palmer B.R., Frampton C.M., Skelton L. (2012). KCNE5 polymorphism rs697829 is associated with QT interval and survival in acute coronary syndromes patients. J Cardiovasc Electrophysiol.

[50] David J.P., Lisewski U., Crump S.M. (2019). Deletion in mice of X-linked, Brugada syndrome- and atrial fibrillation-associated Kcne5 augments ventricular K(V) currents and predisposes to ventricular arrhythmia. FASEB J.

[51] Di Diego J.M., Cordeiro J.M., Goodrow R.J. (2002). Ionic and cellular basis for the predominance of the Brugada syndrome phenotype in males. Circulation.

[52] Millar I.D., Bruce J.I., Brown P.D. (2007). Ion channel diversity, channel expression and function in the choroid plexuses. Cerebrospinal Fluid Res.

[53] Tinel N., Diochot S., Lauritzen I., Barhanin J., Lazdunski M., Borsotto M. (2000). M-type KCNQ2-KCNQ3 potassium channels are modulated by the KCNE2 subunit. FEBS Lett.

[54] Roepke T.K., Kanda V.A., Purtell K., King E.C., Lerner D.J., Abbott G.W. (2011). KCNE2 forms potassium channels with KCNA3 and KCNQ1 in the choroid plexus epithelium. FASEB J.

[55] Kofman O., Sherman W.R., Katz V., Belmaker R.H. (1993). Restoration of brain myo-inositol levels in rats increases latency to lithium-pilocarpine seizures. Psychopharmacology.

[56] Agam G., Shapiro Y., Bersudsky Y., Kofman O., Belmaker R.H. (1994). High-dose peripheral inositol raises brain inositol levels and reverses behavioral effects of inositol depletion by lithium. Pharmacol Biochem Behav.

[57] Abbott G.W., Tai K.K., Neverisky D.L. (2014). KCNQ1, KCNE2, and Na^+^-coupled solute transporters form reciprocally regulating complexes that affect neuronal excitability. Sci Signal.

[58] McCrossan Z.A., Lewis A., Panaghie G. (2003). MinK-related peptide 2 modulates Kv2.1 and Kv3.1 potassium channels in mammalian brain. J Neurosci.

[59] Tam G.W.C., van de Lagemaat L.N., Redon R. (2010). Confirmed rare copy number variants implicate novel genes in schizophrenia. Biochem Soc Trans.

[60] Lussier Y., Fürst O., Fortea E. (2019). Disease-linked mutations alter the stoichiometries of HCN-KCNE2 complexes. Sci Rep.

[61] Singh N.A., Westenskow P., Charlier C. (2003). *KCNQ2* and *KCNQ3* potassium channel genes in benign familial neonatal convulsions: expansion of the functional and mutation spectrum. Brain.

[62] Schroeder B.C., Kubisch C., Stein V., Jentsch T.J. (1998). Moderate loss of function of cyclic-AMP-modulated KCNQ2/KCNQ3 K^+^ channels causes epilepsy. Nature.

[63] Demolombe S., Franco D., de Boer P. (2001). Differential expression of KvLQT1 and its regulator IsK in mouse epithelia. Am J Physiol Cell Physiol.

[64] Vallon V., Grahammer F., Richter K. (2001). Role of KCNE1-dependent K^+^ fluxes in mouse proximal tubule. J Am Soc Nephrol.

[65] Millar I.D., Hartley J.A., Haigh C. (2004). Volume regulation is defective in renal proximal tubule cells isolated from KCNE1 knockout mice. Exp Physiol.

[66] Arrighi I., Bloch-Faure M., Grahammer F. (2001). Altered potassium balance and aldosterone secretion in a mouse model of human congenital long QT syndrome. Proc Natl Acad Sci USA.

[67] Hu Z., Crump S.M., Anand M., Kant R., Levi R., Abbott G.W. (2014). Kcne3 deletion initiates extracardiac arrhythmogenesis in mice. FASEB J.

[68] Talbi K., Ousingsawat J., Centeio R., Schreiber R., Kunzelmann K. (2023). KCNE1 does not shift TMEM16A from a Ca^2+^ dependent to a voltage dependent Cl^–^ channel and is not expressed in renal proximal tubule. Pflügers Archiv.

[69] Palmer L.G., Frindt G. (2007). High-conductance K channels in intercalated cells of the rat distal nephron. Am J Physiol Ren Physiol.

[70] Levy D.I., Wanderling S., Biemesderfer D., Goldstein S.A.N. (2008). MiRP3 acts as an accessory subunit with the BK potassium channel. Am J Physiol Ren Physiol.

[71] Abbott G.W., Butler M.H., Bendahhou S., Dalakas M.C., Ptacek L.J., Goldstein S.A. (2001). MiRP2 forms potassium channels in skeletal muscle with Kv3.4 and is associated with periodic paralysis. Cell.

[72] King E.C., Patel V., Anand M. (2017). Targeted deletion of Kcne3 impairs skeletal muscle function in mice. FASEB J.

[73] Sternberg D., Tabti N., Fournier E., Hainque B., Fontaine B. (2003). Lack of association of the potassium channel-associated peptide MiRP2-R83H variant with periodic paralysis. Neurology.

[74] Jurkat-Rott K., Lehmann-Horn F. (2004). Periodic paralysis mutation MiRP2-R83H in controls: interpretations and general recommendation. Neurology.

[75] Pereira da Silva E.A., Martín-Aragón Baudel M., Navedo M.F., Nieves-Cintrón M. (2022). Ion channel molecular complexes in vascular smooth muscle. Front Physiol.

[76] Abbott G.W., Jepps T.A. (2016). Kcne4 deletion sex-dependently alters vascular reactivity. J Vasc Res.

[77] Villegas-Esguevillas M., Cho S., Vera-Zambrano A. (2023). The novel K(V)_7_ channel activator URO-K10 exerts enhanced pulmonary vascular effects independent of the KCNE4 regulatory subunit. Biomed Pharmacother.

[78] Vetter D.E., Mann J.R., Wangemann P. (1996). Inner ear defects induced by null mutation of the isk gene. Neuron.

[79] Duggal P., Vesely M.R., Wattanasirichaigoon D., Villafane J., Kaushik V., Beggs A.H. (1998). Mutation of the gene for IsK associated with both Jervell and Lange-Nielsen and Romano-Ward forms of Long-QT syndrome. Circulation.

[80] Van Laer L., Carlsson P.I., Ottschytsch N. (2006). The contribution of genes involved in potassium-recycling in the inner ear to noise-induced hearing loss. Hum Mutat.

[81] Oertli A., Rinné S., Moss R. (2021). Molecular mechanism of autosomal recessive long QT-syndrome 1 without deafness. Int J Mol Sci.

[82] Knipper M., Claussen C., Rüttiger L. (2006). Deafness in LIMP2-deficient mice due to early loss of the potassium channel KCNQ1/KCNE1 in marginal cells of the stria vascularis. J Physiol.

[83] Wu X., Zhang L., Li Y. (2021). Gene therapy via canalostomy approach preserves auditory and vestibular functions in a mouse model of Jervell and Lange-Nielsen syndrome type 2. Nat Commun.

[84] Wang W., Kim H.J., Lee J.H. (2014). Functional significance of K^+^ channel β-subunit KCNE3 in auditory neurons. J Biol Chem.

[85] Carricondo F., Romero-Gómez B. (2019). The cochlear spiral ganglion neurons: the auditory portion of the VIII nerve. Anat Rec.

[86] Venglovecz V., Rakonczay Z., Gray M.A., Hegyi P. (2015). Potassium channels in pancreatic duct epithelial cells: their role, function and pathophysiological relevance. Pflügers Arch Eur J Physiol.

[87] Warth R., Garcia Alzamora M., Kim J.K. (2002). The role of KCNQ1/KCNE1 K^+^ channels in intestine and pancreas: lessons from the KCNE1 knockout mouse. Pflügers Archiv.

[88] Roepke T.K., Anantharam A., Kirchhoff P. (2006). The KCNE2 potassium channel ancillary subunit is essential for gastric acid secretion. J Biol Chem.

[89] Lisewski U., Köhncke C., Schleussner L. (2020). Hypochlorhydria reduces mortality in heart failure caused by Kcne2 gene deletion. FASEB J.

[90] Schroeder B.C., Waldegger S., Fehr S. (2000). A constitutively open potassium channel formed by KCNQ1 and KCNE3. Nature.

[91] Preston P., Wartosch L., Günzel D. (2010). Disruption of the K^+^ channel beta-subunit KCNE3 reveals an important role in intestinal and tracheal Cl^–^ transport. J Biol Chem.

[92] Roepke T.K., King E.C., Purtell K., Kanda V.A., Lerner D.J., Abbott G.W. (2011). Genetic dissection reveals unexpected influence of beta subunits on KCNQ1 K^+^ channel polarized trafficking *in vivo*. FASEB J.

[93] Julio-Kalajzić F., Villanueva S., Burgos J. (2018). K(2P) TASK-2 and KCNQ1-KCNE3 K^+^ channels are major players contributing to intestinal anion and fluid secretion. J Physiol.

[94] Alzamora R., O’Mahony F., Bustos V. (2011). Sexual dimorphism and oestrogen regulation of KCNE3 expression modulates the functional properties of KCNQ1 K^+^ channels. J Physiol.

[95] Al-Hazza A., Linley J., Aziz Q., Hunter M., Sandle G. (2016). Upregulation of basolateral small conductance potassium channels (KCNQ1/KCNE3) in ulcerative colitis. Biochem Biophys Res Commun.

[96] Abancens M., Bustos V., Harvey H., McBryan J., Harvey B.J. (2020). Sexual dimorphism in colon cancer. Front Oncol.

[97] Boucher R.C., Stutts M.J., Bromberg P.A., Gatzy J.T. (1981). Regional differences in airway surface liquid composition. J Appl Physiol Respir Environ Exerc Physiol.

[98] Willumsen N.J., Davis C.W., Boucher R.C. (1994). Selective response of human airway epithelia to luminal but not serosal solution hypertonicity. Possible role for proximal airway epithelia as an osmolality transducer. J Clin Investig.

[99] Cotton C.U., Stutts M.J., Knowles M.R., Gatzy J.T., Boucher R.C. (1987). Abnormal apical cell membrane in cystic fibrosis respiratory epithelium. An *in vitro* electrophysiologic analysis. J Clin Investig.

[100] Zhou L., Köhncke C., Hu Z., Roepke T.K., Abbott G.W. (2019). The KCNE2 potassium channel β subunit is required for normal lung function and resilience to ischemia and reperfusion injury. FASEB J.

[101] Hu Z., Kant R., Anand M. (2014). Kcne2 deletion creates a multisystem syndrome predisposing to sudden cardiac death. Circ Cardiovasc Genet.

[102] Roepke T.K., King E.C., Reyna-Neyra A. (2009). Kcne2 deletion uncovers its crucial role in thyroid hormone biosynthesis. Nat Med.

[103] Hu Z., Crump S.M., Zhang P., Abbott G.W. (2016). Kcne2 deletion attenuates acute post-ischaemia/reperfusion myocardial infarction. Cardiovasc Res.

[104] Lee S.M., Nguyen D., Hu Z., Abbott G.W. (2015). Kcne2 deletion promotes atherosclerosis and diet-dependent sudden death. J Mol Cell Cardiol.

[105] Lisewski U., Koehncke C., Wilck N., Buschmeyer B., Pieske B., Roepke T.K. (2016). Increased aldosterone-dependent Kv1.5 recycling predisposes to pacing-induced atrial fibrillation in Kcne3^–/–^ mice. FASEB J.

[106] Liu L., Wang F., Lu H., Ren X., Zou J. (2014). Chromanol 293B, an inhibitor of KCNQ1 channels, enhances glucose-stimulated insulin secretion and increases glucagon-like peptide-1 level in mice. Islets.

[107] Lee S.M., Baik J., Nguyen D. (2017). Kcne2 deletion impairs insulin secretion and causes type 2 diabetes mellitus. FASEB J.

[108] Ullrich S., Su J., Ranta F. (2005). Effects of I(Ks) channel inhibitors in insulin-secreting INS-1 cells. Pflügers Archiv.

[109] Kundu P., Ciobotaru A., Foroughi S., Toro L., Stefani E., Eghbali M. (2008). Hormonal regulation of cardiac KCNE2 gene expression. Mol Cell Endocrinol.

[110] Roepke T.K., Kontogeorgis A., Ovanez C. (2008). Targeted deletion of kcne2 impairs ventricular repolarization via disruption of I(K, slow1) and I(to,f). FASEB J.

[111] Brouillette J., Rivard K., Lizotte E., Fiset C. (2005). Sex and strain differences in adult mouse cardiac repolarization: importance of androgens. Cardiovasc Res.

[112] Karl K., Marcus M. (2002). Electrolyte transport in the mammalian colon: mechanisms and implications for disease. Physiol Rev.

[113] Abbott G.W. (2016). Regulation of human cardiac potassium channels by full-length KCNE3 and KCNE4. Sci Rep.

[114] Deckelbaum R.A., Lobov I.B., Cheung E. (2020). The potassium channel Kcne3 is a *VEGFA*-inducible gene selectively expressed by vascular endothelial tip cells. Angiogenesis.

[115] Zhang Z., Liu S., Wang Z. (2024). Influential upregulation of KCNE4: propelling cancer associated fibroblasts-driven colorectal cancer progression. Cancer Cell Int.

[116] Ohno S., Zankov D.P., Yoshida H. (2007). N- and C-terminal KCNE1 mutations cause distinct phenotypes of long QT syndrome. Heart Rhythm.

[117] Westenskow P., Splawski I., Timothy K.W., Keating M.T., Sanguinetti M.C. (2004). Compound mutations: a common cause of severe long-QT syndrome. Circulation.

[118] Bianchi L., Shen Z., Dennis A.T. (1999). Cellular dysfunction of LQT5-minK mutants: abnormalities of IKs, IKr and trafficking in long QT syndrome. Hum Mol Genet.

[119] Ma L., Lin C., Teng S. (2003). Characterization of a novel Long QT syndrome mutation G52R-KCNE1 in a Chinese family. Cardiovasc Res.

[120] Splawski I., Tristani-Firouzi M., Lehmann M.H., Sanguinetti M.C., Keating M.T. (1997). Mutations in the hminK gene cause long QT syndrome and suppress IKs function. Nat Genet.

[121] Wu D.M., Lai L.P., Zhang M. (2006). Characterization of an LQT5-related mutation in KCNE1, Y81C: implications for a role of KCNE1 cytoplasmic domain in I_Ks_ channel function. Heart Rhythm.

[122] Schulze-Bahr E., Schwarz M., Hauenschild S. (2001). A novel long-QT 5 gene mutation in the C-terminus (V109I) is associated with a mild phenotype. J Mol Med (Berl).

[123] Martinez K., Smith A., Ye D., Zhou W., Tester D.J., Ackerman M.J. (2023). Curcumin, a dietary natural supplement, prolongs the action potential duration of KCNE1-D85N-induced pluripotent stem cell-derived cardiomyocytes. Heart Rhythm.

[124] Yamaguchi Y., Mizumaki K., Hata Y., Inoue H. (2016). Abnormal repolarization dynamics in a patient with KCNE1(G38S) who presented with torsades de pointes. J Electrocardiol.

[125] Yamaguchi Y., Mizumaki K., Hata Y. (2017). Latent pathogenicity of the G38S polymorphism of KCNE1 K^+^ channel modulator. Heart Vessel.

[126] Lane C.M., Giudicessi J.R., Ye D. (2018). Long QT syndrome type 5-Lite: defining the clinical phenotype associated with the potentially proarrhythmic p.Asp85Asn-KCNE1 common genetic variant. Heart Rhythm.

[127] Olesen M.S., Bentzen B.H., Nielsen J.B. (2012). Mutations in the potassium channel subunit KCNE1 are associated with early-onset familial atrial fibrillation. BMC Med Genet.

[128] Ehrlich J.R., Zicha S., Coutu P., Hébert T.E., Nattel S. (2005). Atrial fibrillation-associated minK38G/S polymorphism modulates delayed rectifier current and membrane localization. Cardiovasc Res.

[129] Isbrandt D., Friederich P., Solth A. (2002). Identification and functional characterization of a novel KCNE2 (MiRP1) mutation that alters HERG channel kinetics. J Mol Med (Berl).

[130] Sauer C.W., Marc-Aurele K.L. (2016). A neonate with susceptibility to long QT syndrome type 6 who presented with ventricular fibrillation and sudden unexpected infant death. Am J Case Rep.

[131] Yang Y., Xia M., Jin Q. (2004). Identification of a KCNE2 gain-of-function mutation in patients with familial atrial fibrillation. Am J Hum Genet.

[132] Ohno S., Toyoda F., Zankov D.P. (2009). Novel KCNE3 mutation reduces repolarizing potassium current and associated with long QT syndrome. Hum Mutat.

[133] Zeng Z., Tan C., Teng S. (2007). The single nucleotide polymorphisms of I(Ks) potassium channel genes and their association with atrial fibrillation in a Chinese population. Cardiology.

[134] Hoppe U.C., Marbán E., Johns D.C. (2001). Distinct gene-specific mechanisms of arrhythmia revealed by cardiac gene transfer of two long QT disease genes, *HERG* and KCNE1. Proc Natl Acad Sci USA.

[135] Ackerman M.J., Tester D.J., Jones G.S., Will M.L., Burrow C.R., Curran M.E. (2003). Ethnic differences in cardiac potassium channel variants: implications for genetic susceptibility to sudden cardiac death and genetic testing for congenital long QT syndrome. Mayo Clin Proc.

[136] Paulussen A.D.C., Gilissen R.A.H.J., Armstrong M. (2004). Genetic variations of KCNQ1, KCNH2, SCN5A, KCNE1, and KCNE2 in drug-induced long QT syndrome patients. J Mol Med (Berl).

[137] Nishio Y., Makiyama T., Itoh H. (2009). D85N, a *KCNE1* polymorphism, is a disease-causing gene variant in long QT syndrome. J Am Coll Cardiol.

[138] Friedlander Y., Vatta M., Sotoodehnia N. (2005). Possible association of the human *KCNE1* (minK) gene and QT interval in healthy subjects: evidence from association and linkage analyses in Israeli families. Ann Hum Genet.

[139] Iwasa H., Itoh T., Nagai R., Nakamura Y., Tanaka T. (2000). Twenty single nucleotide polymorphisms (SNPs) and their allelic frequencies in four genes that are responsible for familial long QT syndrome in the Japanese population. J Hum Genet.

[140] Seebohm G., Strutz-Seebohm N., Ureche O.N. (2008). Long QT syndrome-associated mutations in KCNQ1 and KCNE1 subunits disrupt normal endosomal recycling of IKs channels. Circ Res.

[141] Harmer S.C., Wilson A.J., Aldridge R., Tinker A. (2010). Mechanisms of disease pathogenesis in long QT syndrome type 5. Am J Physiol Cell Physiol.

[142] Lin L., Horigome H., Nishigami N., Ohno S., Horie M., Sumazaki R. (2012). Drug-induced QT-interval prolongation and recurrent torsade de pointes in a child with heterotaxy syndrome and KCNE1 D85N polymorphism. J Electrocardiol.

[143] Lopez-Medina A.I., Campos-Staffico A.M., A Chahal C.A. (2024). Genetic risk factors for drug-induced long QT syndrome: findings from a large real-world case-control study. Pharmacogenomics.

[144] Roberts J.D., Krahn A.D., Ackerman M.J. (2017). Loss-of-function KCNE2 variants: true monogenic culprits of long-QT syndrome or proarrhythmic variants requiring secondary provocation?. Circ Arrhythm Electrophysiol.

[145] Ohno S., Toyoda F., Zankov D.P. (2009). Novel KCNE3 mutation reduces repolarizing potassium current and associated with long QT syndrome. Hum Mutat.

[146] Olesen M.S., Bentzen B.H., Nielsen J.B. (2012). Mutations in the potassium channel subunit KCNE1 are associated with early-onset familial atrial fibrillation. BMC Med Genet.

[147] Li L., Shen C., Yao Z., Liang J., Huang C. (2015). Genetic variants of potassium voltage-gated channel genes (KCNQ1, KCNH2, and KCNE1) affected the risk of atrial fibrillation in elderly patients. Genet Test Mol Biomark.

[148] Yao J., Ma Y.T., Xie X., Liu F., Chen B.D. (2012). Association of KCNE1 genetic polymorphisms with atrial fibrillation in a Chinese Han population. Genet Test Mol Biomark.

[149] Miao H., Zhou X., Kabinur K., Zou T., Palida A., Tang B. (2017). Association of *KCNE1* and KCNE4 gene polymorphisms with atrial fibrillation among Uygur and Han Chinese populations in Xinjiang. Zhonghua Yi Xue Yi Chuan Xue Za Zhi.

[150] Miao H., Zhou X., Mao T., Renner W., Abulizi P., Tang B. (2012). Association between KCNE1 (G38S) genetic polymorphism and non-valvular atrial fibrillation in an Uygur population. Wien Klin Wochenschr.

[151] Li Y.Y., Wang L.S., Lu X.Z. (2014). Mink S38G gene polymorphism and atrial fibrillation in the Chinese population: a meta-analysis of 1871 participants. Sci World J.

[152] Akilzhanova A., Rakhimova S., Abilova Z. (2014). Sequence alterations of I(Ks) potassium channel genes in kazakhstani patients with atrial fibrillation. Cent Asian J Global Health.

[153] Wugeti N., Yu-Jun G., Juan S., Mahemuti A. (2015). Correlation analysis between the delayed rectifier potassium channel KCNE1 (G38S) polymorphism and atrial fibrillation among the senior Uygur population in Xinjiang. Genet Mol Res.

[154] Temple J., Frias P., Rottman J. (2005). Atrial fibrillation in KCNE1-null mice. Circ Res.

[155] Prystupa A., Dzida G., Myśliński W., Małaj G., Lorenc T. (2006). MinK gene polymorphism in the pathogenesis of lone atrial fibrillation. Kardiol Pol.

[156] Xu L.X., Yang W.Y., Zhang H.Q., Tao Z.H., Duan C.C. (2008). Study on the correlation between *CETP* TaqIB, *KCNE1* S38G and *eNOS* T-786C gene polymorphisms for predisposition and non-valvular atrial fibrillation. Zhonghua Liuxingbingxue Zazhi.

[157] Husser D., Stridh M., Sörnmo L., Roden D.M., Darbar D., Bollmann A. (2009). A genotype-dependent intermediate ECG phenotype in patients with persistent lone atrial fibrillation genotype ECG-phenotype correlation in atrial fibrillation. Circ Arrhythm Electrophysiol.

[158] Heerdt P.M., Kant R., Hu Z. (2012). Transcriptomic analysis reveals atrial KCNE1 down-regulation following lung lobectomy. J Mol Cell Cardiol.

[159] Voudris K.V., Apostolakis S., Karyofillis P. (2014). Genetic diversity of the KCNE1 gene and susceptibility to postoperative atrial fibrillation. Am Heart J.

[160] Pérez-Hernández M., Matamoros M., Barana A. (2016). Pitx2c increases in atrial myocytes from chronic atrial fibrillation patients enhancing IKs and decreasing ICa, L. Cardiovasc Res.

[161] Nielsen J.B., Bentzen B.H., Olesen M.S. (2014). Gain-of-function mutations in potassium channel subunit KCNE2 associated with early-onset lone atrial fibrillation. Biomarkers Med.

[162] Liu X., Li Y., Zhang H., Ji Y., Zhao Z., Wang C. (2019). The research of ion channel-related gene polymorphisms with atrial fibrillation in the Chinese Han population. Mol Genet Genomic Med.

[163] Lundby A., Ravn L.S., Svendsen J.H., Hauns S., Olesen S.P., Schmitt N. (2008). KCNE3 mutation V17M identified in a patient with lone atrial fibrillation. Cell Physiol Biochem.

[164] Wu J., Shimizu W., Ding W.G. (2010). KCNE2 modulation of Kv4.3 current and its potential role in fatal rhythm disorders. Heart Rhythm.

[165] Delpón E., Cordeiro J.M., Núñez L. (2008). Functional effects of KCNE3 mutation and its role in the development of Brugada syndrome. Circ Arrhythm Electrophysiol.

[166] Nakajima T., Wu J., Kaneko Y. (2012). KCNE3 T4A as the genetic basis of Brugada-pattern electrocardiogram. Circ J.

[167] Fatini C., Sticchi E., Marcucci R. (2010). S38G single-nucleotide polymorphism at the KCNE1 locus is associated with heart failure. Heart Rhythm.

[168] Abbott G.W., Butler M.H., Goldstein S.A.N. (2006). Phosphorylation and protonation of neighboring MiRP2 sites: function and pathophysiology of MiRP2-Kv3.4 potassium channels in periodic paralysis. FASEB J.

[169] Dias Da Silva M.R., Cerutti J.M., Arnaldi L.A.T., Maciel R.M.B. (2002). A mutation in the *KCNE3* potassium channel gene is associated with susceptibility to thyrotoxic hypokalemic periodic paralysis. J Clin Endocrinol Metab.

[170] Purtell K., Paroder-Belenitsky M., Reyna-Neyra A. (2012). The KCNQ1-KCNE2 K^+^ channel is required for adequate thyroid I^-^ uptake. FASEB J.

[171] Pan Y., Zhao L., Liu Z. (2007). *KCNE2* a down-regulated gene identified by in silico analysis, suppressed proliferation of gastric cancer cells. Cancer Lett.

[172] Salsbury G., Cambridge E.L., McIntyre Z. (2014). Disruption of the potassium channel regulatory subunit KCNE2 causes iron-deficient anemia. Exp Hematol.

[173] Tian K., Tao Z., Chen Y. (2023). KCNE4 expression is correlated with the pathological characteristics of colorectal cancer patients and associated with the radioresistance of cancer cells. Pathol Res Pract.

[174] Lee S.M., Nguyen D., Anand M. (2016). Kcne2 deletion causes early-onset nonalcoholic fatty liver disease via iron deficiency anemia. Sci Rep.

[175] Hu Z., Jepps T.A., Zhou L., Liu J., Li M., Abbott G.W. (2019). Kcne4 deletion sex dependently inhibits the RISK pathway response and exacerbates hepatic ischemia-reperfusion injury in mice. Am J Physiol Regul Integr Comp Physiol.

[176] Mondéjar-Parreño G., Barreira B., Callejo M. (2020). Uncovered contribution of Kv7 channels to pulmonary vascular tone in pulmonary arterial hypertension. Hypertension.

[177] Eun C., Geoffrey A. (2007). The MiRP2-Kv3.4 potassium channel: muscling in on Alzheimer’s disease. Mol Pharmacol.

[178] de Jong I.E.M., Jepps T.A. (2018). Impaired Kv7 channel function in cerebral arteries of a tauopathy mouse model (rTg4510). Phys Rep.

[179] Doi K., Sato T., Kuramasu T. (2005). Ménière’s disease is associated with single nucleotide polymorphisms in the human potassium channel genes, *KCNE1* and KCNE3. ORL J Otorhinolaryngol Relat Spec.

[180] Campbell C.A., Della Santina C.C., Meyer N.C. (2010). Polymorphisms in KCNE1 or KCNE3 are not associated with Ménière disease in the Caucasian population. Am J Med Genet A.

[181] Dai Q., Wang D., Zheng H. (2019). The polymorphic analysis of the human potassium channel KCNE gene family in Meniere’s disease - a preliminary study. J Int Adv Otol.

[182] Mano R., Tanaka T., Hashiguchi S. (2022). Induction of potassium channel regulator KCNE4 in a submandibular lymph node metastasis model. Sci Rep.

[183] Tsevi I., Vicente R., Grande M. (2005). KCNQ1/KCNE1 channels during germ-cell differentiation in the rat: expression associated with testis pathologies. J Cell Physiol.

